# Global expression studies in baker's yeast reveal target genes for the improvement of industrially-relevant traits: the cases of *CAF16 *and *ORC2*

**DOI:** 10.1186/1475-2859-9-56

**Published:** 2010-07-13

**Authors:** Roberto Pérez-Torrado, Joaquín Panadero, María José Hernández-López, José Antonio Prieto, Francisca Randez-Gil

**Affiliations:** 1 Department of Biotechnology, Instituto de Agroquímica y Tecnología de los Alimentos (CSIC), P.O. Box 73, E-46100-Burjassot Valencia, Spain

## Abstract

**Background:**

Recent years have seen a huge growth in the market of industrial yeasts with the need for strains affording better performance or to be used in new applications. Stress tolerance of commercial *Saccharomyces cerevisiae *yeasts is, without doubt, a trait that needs improving. Such trait is, however, complex, and therefore only in-depth knowledge of their biochemical, physiological and genetic principles can help us to define improvement strategies and to identify the key factors for strain selection.

**Results:**

We have determined the transcriptional response of commercial baker's yeast cells to both high-sucrose and lean dough by using DNA macroarrays and liquid dough (LD) model system. Cells from compressed yeast blocks display a reciprocal transcription program to that commonly reported for laboratory strains exposed to osmotic stress. This discrepancy likely reflects differences in strain background and/or experimental design. Quite remarkably, we also found that the transcriptional response of starved baker's yeast cells was qualitatively similar in the presence or absence of sucrose in the LD. Nevertheless, there was a set of differentially regulated genes, which might be relevant for cells to adapt to high osmolarity. Consistent with this, overexpression of *CAF16 *or *ORC2*, two transcriptional factor-encoding genes included in this group, had positive effects on leavening activity of baker's yeast. Moreover, these effects were more pronounced during freezing and frozen storage of high-sucrose LD.

**Conclusions:**

Engineering of differentially regulated genes opens the possibility to improve the physiological behavior of baker's yeast cells under stress conditions like those encountered in downstream applications.

## Background

Variation in the osmotic pressure surrounding yeast occurs constantly in almost all steps from biomass production to bread making [1-3]. This is especially true for sweet bread products, which contain high amounts of sucrose or glucose/fructose syrup added as sweetener. Sweet dough exerts strong osmotic stress on yeasts, seriously affecting their fermentative capacity. Consequently, proofing times are longer for sweet bakery loaves and yield low volume products. To face these problems, manufacturers use greater amounts of yeast in the dough formulation; however, this is expensive and final taste and texture are suboptimal. Therefore, there is a great interest in developing new baker's yeast strains with improved osmotic resistance.

The osmotic response of *Saccharomyces cerevisiae *has been well characterized in laboratory strains [4]. Exposure of yeast cells to highly osmotic environments provoke the up-regulation of ca. 400 genes, covering a wide variety of physiological functions, including carbon and amino acid metabolism, redox balance, anti-oxidant protection, ATPases, membrane proteins, chaperones, cytoskeletal and cell wall adaptations [5-7]. Such information has helped identify target genes, regulators and pathways involved in osmotic response [3]. However, given the special characteristics of these strains, it is questionable whether such data can be used to develop molecular strategies to improve osmotic stress resistance in industrial yeasts.

Commercial baker's yeasts are domesticated strains of *S. cerevisiae*, selected and optimized for baking applications. Most of them are homothallic, with a high and irregular degree of ploidy and low sporulation ability [8]. Moreover, they exhibit chromosomal-length polymorphisms and rearranged chromosomes with multiple translocations. Thus, baker's yeast strains differ genomically from other *S. cerevisiae *strains in their adaptation to industrial conditions [3]. Their ability to ferment bread dough efficiently and behavior under stress conditions could also reflect specific expression patterns, as has been documented for wine strains [9,10]. Therefore, applying functional genomics to commercial strains under industrial conditions is a clue to understanding their osmotic adaptation mechanisms, thus enabling the design of new approaches to genetic improvement.

In this work, we have used compressed yeast blocks and flour-free liquid dough (LD) model system [11], to study the transcriptional response of industrial yeast to high-sucrose in bread dough. The aim was to reproduce as much as possible the conditions in which bakers employ commercial yeast, quite often as compressed yeast, in order to identify the set of genes regulated under these conditions. Overall, our work provides new insights into the links between technological traits and genetic determinants, showing the potential of this approach for genetic engineering of bakers' yeast.

## Results

We compared the genome-wide transcription pattern of starved cells from compressed yeast blocks with that of cells cultured for 60 min in high-sugar liquid dough (LD). In previous studies, we demonstrated that this flour-free model system mimics the nutritional and stressful environment encountered by baker's yeast cells in bread dough [11]. Furthermore, data obtained from the macroarray analysis agreed very well with Northern blot data for five marker genes (additional file 1): two induced, *PIS1 *(2.7-fold induction on gene filter and 1.6-fold induction in Northerns) and *PHO3 *(7.3/4.0), and three repressed, *OLE1 *(2.7-fold repression on gene filter and 1.7-fold repression in Northerns), *HSP12 *(4.7/4.1) and *HSP26 *(4.0/3.1). Hence, the combination of LD model system and gene filters is an appropriate and simple way to perform genome-wide transcriptional analysis under commercially important, but experimentally intractable, conditions.

### Global expression after transfer to high-sucrose LD: overview

**Figure 1:**
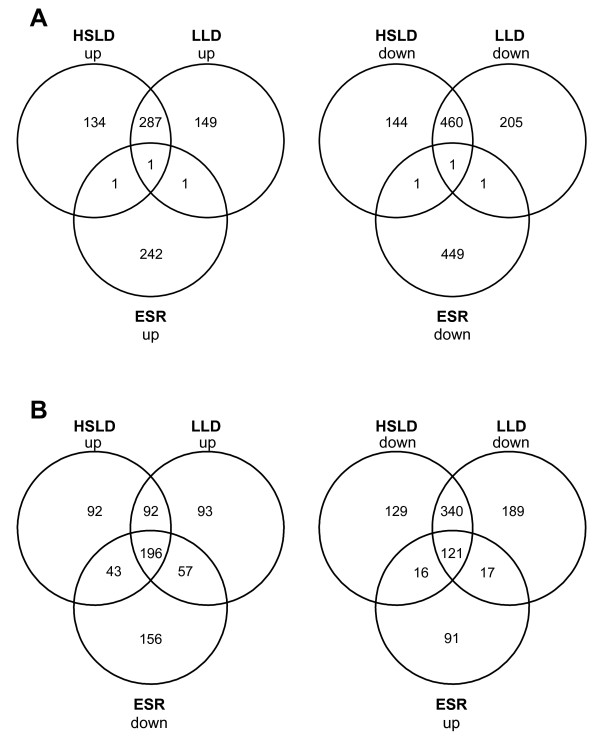
**Comparison of the environmental stress (ESR), lean (LLD) and high-sucrose liquid dough (HSLD) responses**. The set of genes up- (up) or down-regulated (down) 4.0-fold or more (log_2 _ratio of ± 2) after 60 min of transfer of yeast cells to high-sucrose LD (HSLD) or lean LD (LLD) (see additional file 2), was compared with the ESR genes identified by Gasch *et al*. [6], and the number of genes in common are shown in Venn diagrams for both the induced and repressed genes in each case. A) Up- (left) and down-regulated (right) genes in HSLD and LLD were compared with up- and down-regulated ERS genes, respectively. B) Up- (left) and down-regulated (right) genes in HSLD and LLD were compared with down- and up-regulated ERS genes, respectively.

Macroarray data revealed that the shift of starved cells to high-sucrose LD greatly affected the gene expression program of baker's yeast. The list of genes induced or repressed 4.0-fold or more (log_2 _ratio of ±2) is available as additional file 2. Significant variations in the mRNA levels of 1,029 genes, 423 induced and 606 repressed, were observed. The list of regulated genes was screened for enrichment of specific functional categories (Table 1), from which several general conclusions can be drawn. First, the observed expression changes mainly reflect the suppression of starvation conditions in compressed baker's yeast cells, rather than exposure to a hostile medium. Thus, a significant number of genes encoding ribosomal proteins or involved in protein biosynthesis and ribosomal processing, functions known to be transiently repressed by stress conditions [5,6], were significantly up-regulated (Table 1). On the contrary, genes associated with GO biological processes as response to stress, i.e. *TPS1*, *TSL1*, *CTT1*, *GLO1*, *HSP12*, *HSP26, HSP30 *or *GRE1*, that are normally induced by global stress responses [5,6], tended to be repressed (Table 1). Such reciprocal expression pattern has been reported previously when yeast cells were subjected to opposite stresses [6]. This view was confirmed by comparing the dataset of genes differentially regulated in our study, with the list of ESR (Environmental Stress Response) genes, a set of genes showing a common transcriptional response to different stress conditions [6]. As can be seen, only 4 genes showed a common response (Figure 1A). On the contrary, 239 and 137 genes, induced and repressed in high-sucrose LD, respectively, displayed an opposite transcriptional response in the ESR (Figure 1B). Second, enriched GO terms are mainly associated with energy generation and metabolic functions. Indeed, aerobic respiration, tricarboxilic acid cycle, oxidative phosphorylation or fatty acid oxidation were strongly overrepresented among the genes that were repressed (Table 1), reflecting a shift from carbon starvation conditions to active fermentation. Third, the transcriptional response after 60 min of inoculation of baker's yeast cells also reflects the variation in environmental conditions and/or nutrient concentration during the fermentation process. For instance, we noted a significant induction of *THI6*, *THI7*, *THI21*, *THI22*, *THI80 *and *PHO3*, all of them involved in thiamine biosynthesis or transport under anaerobic conditions (additional file 2). On the contrary, *THI5*, *THI11 *and *THI12*, encoding biosynthetic enzymes involved in the production of thiamine under aerobic conditions, were repressed. This clearly reflects the microaerophilic conditions found by yeast cells in regular bread dough.

**Table 1:** Enriched GO categories for up- and down-regulated genes at 60 min after onset of fermentation of baker's yeast cells in high-sucrose LD.

Functional Group	n	Functional Group	n
**Up (423 genes)**		**Down (606 genes)**	
Ribosome (SCE03010 p = 1.5E-59)	78	Cellular carbohydrate metabolic process	57
Translation (GO0006412 p = 6.2E-09)	113	(GO0044262 p = 2.1E-12)	
Ribonucleoside monophosphate metabolic process	10	Aerobic respiration (GO0009060 p = 9.5E-07)	25
(GO 0009161) p = 8.1E-05)		Response to stress (GO006950 p = 9.8E-05)	16
tRNA metabolic process (GO0006399 p = 2.1E-03)	21	Starch and sucrose metabolism	18
Nuclear transport (GO0051169 p = 1.2E-03)	20	(Kegg pathway p = 7.7E-05)	
RNA helicase activity (GO003724 p = 1.2E-03)	10	Oxidative phosphorylation (GO0006119 p = 2.9E-04)	16
		Tricarboxylic acid cycle (GO0006099 p = 1.8E-04)	10
		Coenzyme metabolic process (GO0006732 p = 1.0E-04)	32
		Response to water deprivation (GO0009414 p = 9.0E-03)	4
		Water soluble vitamin metabolic process	18
		(GO0006767 p = 3.7E-03)	
		Carbohydrate transport (GO0008643 p = 3.1E-03)	12
		Fatty acid oxidation (GO0019305 p = 1.1E-03)	6

**n**, Number of genes found in the indicated category

### Differences in the global transcriptional response between lean and high-sucrose dough

The transcriptional response of commercial baker's yeast cells in lean and high-sucrose LD were compared. Additional file 2 provides the complete list of genes showing at least 4-fold up- or down-regulation (log_2 _ratio of ±2) at 60 min after the transfer of starved cells from compressed yeast blocks to lean LD. A total of 1,105 ORFs showed significant expression changes, being 438 induced and 667 repressed. Again, comparison of regulated genes in lean LD with the list of ERS genes revealed the lack of common responses (Figure 1). Moreover, the list of enriched GO terms (Table 2) was similar to that observed in cells transferred by 60 min to high-sucrose LD (Table 1). Only a few functional categories, comprising a small number of genes were differentially over-represented in lean LD (Table 2). Consistent with this, direct comparison of the set of regulated genes in lean and high-sucrose LD, showed again a high similarity. In this analysis, genes with no reliable data in any of the two conditions were excluded. Of 423 and 606 genes up- and down-regulated in high-sucrose, 288 (68%) and 461 (76%) genes, followed the same change in lean LD. Moreover, there was none ORF showing reverse regulation. The complete list of commonly regulated genes is also shown in additional file 2.

**Table 2:** Enriched GO categories for up- and down-regulated genes at 60 min after onset of fermentation of baker's yeast cells in lean LD.

Functional Group	n	Functional Group	n
**Up (438 genes)**		**Down (667 genes)**	
Ribosome (SCE03010 p = 3.8E-64)	81	Aerobic respiration (GO0009060 p = 2.1E-06)	26
Translation (GO0006412 p = 1.5E-47)	124	Oxidative phosphorylation (GO0006119 p = 9.9E-04)	16
Ribonucleoside monophosphate metabolic process	8	Tricarboxylic acid cycle (GO0006099 p = 4.4E-04)	10
(GO 0009161) p = 2.8E-03)		Starch and sucrose metabolism (Kegg path. p = 1.4E-04)	17
tRNA metabolic process (GO0006399 p = 3.9E-04)	23	Response to external stimulus (GO0009605 p = 6.1E-03)	10
Nuclear transport (GO0051169 p = 1.8E-04)	22	Carbohydrate transport (GO0008643 p = 2.5E-03)	13
RNA helicase activity (GO003724 p = 5.1E-03)	9	Coenzyme catabolic process (GO0009109 p = 1.7E-03)	33
snRNA modification (GO00 p = 2.9E-04)	5		

**n**, Number of genes found in the indicated category

### Genes differentially regulated in high-sucrose

We looked more in detail at those genes that were differentially regulated by the presence of sucrose in the LD system. A total of 135 and 145 ORFs, showing no response in lean LD were significantly induced and repressed, respectively (additional file 2). There were no enriched GO terms among the down-regulated genes. However, the list of induced genes showed prevalent functional categories including ribosome biogenesis and assembly (GO0005840 p = 6.7E-12 n = 32), tRNA metabolic process (GO0006399 p = 4.8E-03 n = 10) and snRNA modification (GO00040031 p = 6.7E-03 n = 3). Furthermore, the list of up-regulated genes included six genes encoding proteins with regulatory functions on transcription and/or translation, *CAF16*, *HAL9*, *MED4*, *SWC4*, *ORC2 *and *TAF1*. All of them have been reported to be induced by osmotic stress conditions [5], and two, *CAF16 *and *ORC2 *have been additionally identified as cold-regulated [12]. These observations suggest the need for a remodeling of the transcriptional and translational machinery under extreme osmolarity conditions.

### LD-induced genes versus HST genes

**Figure 2:**
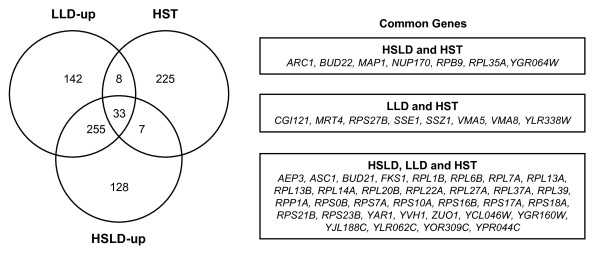
**Comparison of the genes specifically or commonly up-regulated after 60 min of transfer to high-sucrose and lean LD with the genes identified as required for tolerance to high sucrose**. The Venn diagram shows the number of genes up-regulated in high-sucrose (HSLD) and lean (LLD) LD (additional file 2) that are common to the high-sucrose tolerance (HST) dataset (273 genes) identified by Ando *et al*. [13].

We next compared the list of genes specifically or commonly up-regulated in LD under lean and high-sucrose conditions with the set of genes identified by Ando *et al*. [13], concretely 273 genes, as required for tolerance to high sucrose (HST) in a genome-wide screening of *S. cerevisiae *deletion strains (Figure 2). The reason for do this was that in this study, as much as 269 from 273 genes showed cross-sensitivities to sorbitol and NaCl [13]. Consequently, the expression of these essential genes might be expected to be affected in either high-sucrose or lean dough. Seven genes were found in common exclusively between high-sugar LD and HST set of genes (Figure 2). We next compared the list of HST genes with that of 150 genes specifically up-regulated in lean LD and 288 genes that were commonly induced in both high-sugar and lean LD (additional file 2). The comparison revealed a further overlap of 8 and 33 genes among those specifically induced in lean LD and co-induced in both LD systems, respectively (Figure 2). Of these 48 genes found in any condition, 24 genes (50%) encode proteins involved in translation (GO0006412 p = 5.8E-11). Hence, our results make again emphasis in the importance of modulating the transcript levels for genes encoding the translation apparatus and its regulators.

### Overexpression of *CAF16 *and *ORC2 *enhances baking performance of yeast cells exposed to osmotic and freeze stress

We addressed the question of whether gene filter data might reveal target genes for strain selection. We rationalized that products of genes differentially regulated in the LD system (additional file 2) could be important for yeast cells to adapt to extremely low water activity conditions. Eleven genes displaying a significant induction at 60 min after inoculation of commercial baker's yeast cells in LD were tested. Of them, 6 genes (*CAF130*, *CDC10*, *FUR1*, *SEC14*, *YVH1 *and *ZUO1*) showed to be commonly up-regulated in lean and high-sucrose LD, while 5 genes (*CAF16*, *MFT1*, *NMT1*, *ORC2 *and *SSF2*) were specifically induced in high-sucrose (additional file 2).

**Figure 3:**
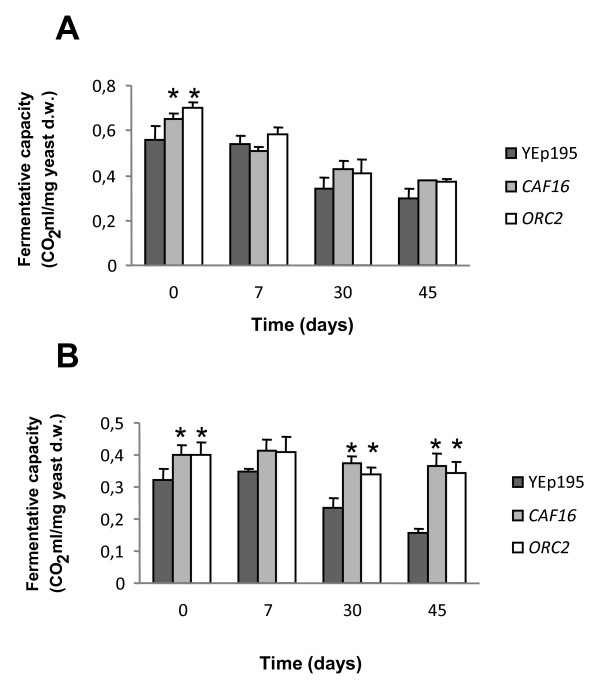
**Fermentative capacity of *CAF16*- and *ORC2*-overexpressing strains**. A) Molasses-plate grown cells of the HS13 baker's yeast strain transformed with the plasmids YEplac195 (control, black bars, YEp195), YEpCAF16 (grey bars) and YEpORC2 (white bars) were used to prepare lean liquid dough (LD) samples as described in the Materials and Methods section. Samples were then quickly frozen at -80°C for 1 h and stored at -20°C. At the indicated times, the frozen LD was left to thaw at 30°C and CO_2 _production was recorded. Values are expressed as ml of CO_2 _produced for 180 min of fermentation and represent the means of at least three independent experiments. B) 25% sucrose-containing LD samples were analyzed as above. *, *P *< 0.05 for gas production of the overexpressing strain compared to gas production of the control strain.

The selected genes were cloned into the shuttle vector YEplac195 and the resulting plasmids were used to transform the industrial HS13 baker's yeast strain (Ura^-^). Transformants were selected by auxotrophic complementation and the functionality of the recombinant strains analyzed for testing their ability to produce CO_2 _in high-sucrose LD. However, only overexpression of *CAF16 *and *ORC2*, two of the six transcriptional factor-encoding genes [14-16], identified as specifically induced in high-sucrose LD (additional file 2), had significant positive effects on leavening activity of baker's yeast cells (Table 3 and Figure 3, control, 0 days). Because of this, only strains overexpressing these two genes were further characterized.

**Table 3:** CO_2 _production by different HS13 transformants^a^

	**ml CO**_**2**_**/mg yeast (d.w.) ± SD (*P *value)**	
	
Plasmad	20% Sucrose	30% Sucrose
YEplac195	0.401 ± 0.005	0.255 ± 0.003
YEpCAF130	0.385 ± 0.015 (0.218)	nd
YEpCDC10	0.425 ± 0.017 (0.140)	nd
YEpFUR1	0.401 ± 0.009 (0.137)	nd
YEpSEC14	0.422 ± 0.022 (0.112)	nd
YEpYVH1	0.383 ± 0.013 (0.095)	nd
YEpZUO1	0.435 ± 0.019 (0.110)	nd
YEpCAF16	0.462 ± 0.012 (0.017)*	0.400 ± 0.030 (0.003)*
YEpMFT1	0.440 ± 0.030 (0.155)	nd
YEpNMT1	0.426 ± 0.022 (0.204)	nd
YEpORC2	0.446 ± 0.018 (0.022)*	0.402 ± 0.036 (0.012)*
YEpSSF2	0.385 ± 0.015 (0.175)	nd

^a^Cells were grown on molasses plates and tested for gas production as described in the Materials and Methods section. Values represent the mean of at least three independent experiments. SD, standard deviation. nd, non-determined. *, *P *< 0.05 for gas production of the overexpressing strain compared to gas production of the control strain (YEplac195).

Thus, we then inspected the behavior of the recombinant strains in frozen products. Freezing is a complex stress in which cellular damage by osmotic shrinkage is one of the main causes of death [17,18]. As can be seen in Figure 3A, CO_2 _production during frozen storage of lean LD was almost similar for all the strains. However, overexpression of *CAF16 *or *ORC2 *appeared to play a stress-protective role in high-sucrose LD (Figure 3B). Indeed, CO_2 _production attained by overexpression of either of these genes in 45-day pre-frozen high-sucrose LD was about 2-fold higher than that observed with the control strain (Figure 3B).

Finally, YEpCAF16 and YEpORC2 transformants were analyzed for growth in molasses medium or in high-sucrose LD (Table 4). A good behavior in these media is critical for the technological applicability of industrial strains [1-3]. As can be seen, neither high copy number of *CAF16 *nor *ORC2 *altered the specific growth rate under the assay conditions (Table 4).

**Table 4:** Specific growth rate of HS13 transformants

	**μ**_**max **_**(h**^**-1**^**)**^***a***^** ± SD**	
	
Plasmad	HSLD	Molasses
YEplac195	0.21 ± 0.02	0.51 ± 0.03
YEpCAF16	0.19 ± 0.02	0.50 ± 0.02
YEpORC2	0.22 ± 0.04	0.50 ± 0.03

^a^Cells were grown in high-sucrose liquid dough (HSLD) or molasses medium. Values represent the mean of at least three independent experiments. SD, standard deviation. μ_max _was calculated as described in the Materials and Methods section.

## Discussion

Here we show relevant information concerning the transcriptional response of commercial baker's yeast in high-sucrose and lean dough. We demonstrated that cells from compressed yeast blocks, the main source of fresh baker's yeast, display a reciprocal transcription program to that commonly reported for laboratory strains exposed to high-osmotic conditions [5,6]. This discrepancy likely reflects differences in strain background and/or experimental design. Indeed, most or all commercial baker's yeasts are poliploid or aneuploid strains with approximately 3n-4n DNA content [19,20]. Moreover, we used starved yeast cells, as do the bakers in the bread-making process, in contrast to the YPD-grown early-log phase cells of laboratory strains examined in previous works [5-7]. Overall, these data further support the idea that studies involving industrial yeast should be performed under those particular conditions encountered in downstream applications, as it has been previously suggested [1-3,20].

We found that the transcriptional response of starved baker's yeast cells was qualitatively similar in the presence or absence of sucrose in the LD model system. This was, in some way, not surprising since a previous study by Causton *et al*. [5] had shown that the genetic response to high salt and to sorbitol are remarkably similar to each other. Cells inoculated in either lean or high-sucrose LD are indeed exposed to the same nutrient environment, except for sucrose. As a result, water activity is low in both systems (*a*_w _= 0.97 and 0.95, respectively). Moreover, a recent survey for sucrose-sensitivity mutants revealed that 269 of 273 genes identified as required under these conditions (High Sucrose Tolerance, HST genes), showed cross-sensitivities to sorbitol and NaCl [13]. Thus, decreased water activity appears to be a main drive force of the transcriptional program triggered by yeast cells in bread dough. In this line, our study has identified genes that might be important to cope with high-osmolarity. Indeed, 48 genes found as specifically or commonly induced in high-sucrose and lean LD overlap with genes reported as essential in the HST analysis [13]. Of them, 50% encode proteins involved in or related with translation. Osmotic stress is generally considered to be a growth-restrictive condition and regulation of genes involved in protein biosynthesis constitutes an important part of the overall adaptive response in *S. cerevisiae *[7,21].

Our study also revealed the existence of genes differentially regulated in high-sucrose LD, whose activity might be important for the adaptive response of yeast cells to severe osmotic stress. In particular, engineering of genes with regulatory functions has the potential to affect simultaneously the activity of many cellular functions. In this line, previous work had shown that overexpression of the calcineurin-target *CRZ1 *and unrelated genes encoding Cys_2_/His_2_-type zinc finger proteins confers freeze tolerance and enhances fermentative capacity of baker's yeast [22].

Six genes with these characteristics, *CAF16*, *HAL9*, *MED4*, *SWC4*, *ORC2 *and *TAF1*, were found from the list of 135 genes specifically up-regulated in high-sucrose LD. Two of them, *CAF16 *and *ORC2 *were tested together with other nine genes for their ability to provide enhanced fermentative performance to baker's yeast cells. Quite remarkably, both *CAF16 *and *ORC2 *have been reported to be induced by either, osmotic [5] and cold stress [12]. In agreement with this, we found that transformants of the industrial HS13 strain, in which the *CAF16 *or *ORC2 *genes had been introduced in a high-copy number, exhibited increased metabolic activity in LD. This was especially true in frozen high-sucrose LD, where the presence of salt (around 2%, flour basis), combined with sucrose and ice-crystal formation exposes yeast cells to both ionic and osmotic stress [22,23]. Indeed, ionic imbalance caused by ice-crystal formation is an important factor determining freeze injury in all living organisms [24]. Thus, enhanced expression of *CAF16 *and *ORC2 *would allow yeast cells to alleviate the harmful effects of ionic stress during freezing. *CAF16 *is a member of the non-transporter group of the ATP-binding cassette (ABC) superfamily [25], and component of the Ccr4-Not transcriptional regulatory complex [14]. In the case of environmental stress, it has been described that this complex affects the Msn2p-dependent transcriptional activation [26,27], and the stress-specific response modulated by the transcription factor Skn7p [28]. On the other hand, Orc2p is a subunit of the origin recognition complex (ORC) that functions in pre-replication complex formation [29] and in chromatin silencing at telomere [16]. Interestingly, Orc2p also functions in the transcriptional regulation of stress-responsive genes, acting as a repressor [30,31] or an activator able to induce expression of highly transcribed genes positioned nearby ORCs [15]. Nevertheless, more work is needed to establish the functional connections between overexpression of *CAF16 *and *ORC2 *and ion and freeze tolerance in high-sucrose dough.

## Conclusions

Global transcriptional approach is a powerful tool to overcome yeast response studies under complex conditions. Such information is clearly necessary in establishing the relationship between genetic determinants and industrial traits and in defining targets for strain selection and improvement. Thus, the finding that overexpression of *CAF16 *and *ORC2 *helps yeast cells to face with osmotic and freeze stress, underlies the importance of this strategy and opens the possibility to new advances. Indeed, other genes determining the same phenotypic character might be also found from the list of regulated genes. If confirmed, the use of engineered strains for these genes may be a way to optimize gassing rate in both fresh and frozen high-sucrose dough, leading to bakery products with lower cost and better organoleptic properties.

## Materials and methods

### Strains, culture media and general methods

The baker's yeast strains L'Hirondelle and HS13 were used throughout this work. HS13 is a uracil auxotrophic non-commercial strain (Lesaffre International, Lille, France), while L'Hirondelle is a commercial strain produced by the Lesaffre Group and usually employed for general baking. The *E. coli *strain DH10B was used as the host for plasmid construction. Yeast cells were cultured at 30°C in defined media, SD (0.2% yeast nitrogen base without amino acids [DIFCO], 0.5% (NH_4_)_2_SO_4_, 2% glucose). *E. coli *was grown in Luria Bertani (LB) medium (1% peptone, 0.5% yeast extract, 0.5% NaCl) supplemented with ampicillin (50 mg/l). Yeast cells were transformed by the lithium acetate method [32], and transformants were selected by auxotrophic complementation in SD plates. *E. coli *was transformed by electroporation following the manufacturer's instructions (Eppendorf AG, Hamburg, Germany).

### Yeast biomass preparation

Compressed yeast packs were acquired from a local distributor, maintained at 4°C, and used no longer than 5 days after the production date. Weighed samples were resuspended in 4°C distilled water containing 27 g/l NaCl, vortexed, and the OD_600 _of the resulting suspension was measured. Final yeast concentration was adjusted to approximately 15 mg (dry weight) per ml. To do this, cell mass was related to optical density measurements, OD_600 _= 1 equals 0.35 mg cells dry weight/ml [11].

Yeast biomass from HS13 transformants was prepared by cultivating cells (7.6 units of OD_600_) on molasses (5.0 g beet molasses [49% sucrose], 0.5 g (NH_4_)_2_HPO_4_, 26.0 g agar and 20 μg biotin per liter; pH 5.0) plates (140-mm diameter) for 20 h at 30°C. Then, yeast cells were recovered by washing the plate surface with 2 × 10 ml of distilled water and the yeast suspension was poured into a tube. After centrifugation, the yeast cake was washed twice with distilled water (4°C), resuspended in saline solution and the final yeast concentration was adjusted as above for further analysis of CO_2 _production.

### Liquid dough (LD) model system

High-sucrose and lean LD solutions were prepared as previously described [11]. Briefly, a 5 × concentrated nutrient solution, containing 5 g MgSO_4_·7H_2_O, 2 g KCl, 11.75 g (NH_4_)_2_HPO_4_, 4 mg thiamine, 4 mg pyridoxine, and 40 mg nicotinic acid in a final volume of 250 ml of 0.75 M citrate buffer (pH 5.5), was prepared. Twenty ml of the concentrated nutrient solution was added to a tube containing 0.5 g yeast extract, 3 g glucose, 9 g maltose, 12 g sorbitol and 50 g sucrose, and the mixture dissolved by sonication. Distilled water was added to a final volume of 100 ml, and the solution filter-sterilized. Lean LD was prepared as above, except that no sucrose was added.

### Gas production measurements

Fifteen ml of yeast suspension was poured into a 250-ml screw cap graduated bottle, placed in a 30°C water bath and gently shaken (80 rpm). After 15 min, 15 ml of 30°C pre-warmed LD was added and the amount of CO_2 _evolved recorded in a Fermograph II (ATTO Co., Ltd., Tokyo, Japan). Samples for freezing were kept at -80°C for 1 h and then stored at -20°C. At different times, they were thawed at 30°C for 30 min before measuring gassing power. In all cases, CO_2 _production was recorded for 180 min. Values are expressed as ml of CO_2 _per mg of yeast cells, dry weight.

### Growth rate estimation

Culture growth was followed by measuring OD_600 _with a Polarstar plate spectrophotometer in six replicates of 225 μl volumes for high-sucrose LD and molasses medium. Maximum specific growth rate was calculated from each condition by directly fitting OD_600_/ml versus time to the reparametrized Gompertz equation proposed by Zwietering *et al*. [33]: y = D*exp{-exp[((μmax*e)/*D*)*(λ - *t*))+1] where y = ln(Nt/N_0_), N_0 _is the initial population (OD_600_/ml) and Nt is the population at time *t*; *D *= ln(N∞/N_0_) is the maximum population value reached with N∞ as the asymptotic maximum, μmax is the maximum specific growth rate (h_-__1_), and λ the lag phase period (h). Growth data from each treatment and yeast were fitted by a non-linear regression procedure, minimizing the sum of squares of the difference between experimental data and the fitted model, i.e., loss function (observed-predicted). This task was accomplished using the non-linear module of the Statistica 6.0 software package and its Quasi-Newton option.

### Plasmids

PCR-amplified fragments containing the whole sequence of *CAF130*, *CDC10*, *FUR1*, *SEC14*, *YVH1*, *ZUO1*, *CAF16*, *MFT1*, *NMT1*, *ORC2 *and *SSF2 *gene, including its own promoter and terminator were obtained with specific synthetic oligonucleotides (additional file 3). The corresponding fragments were digested with the appropriate set of enzymes, XbaI/PstI (*CAF16*, *CDC10*, *NMT1*, *ORC2*, *SEC14*, *ZUO1*), SalI/HindIII (*CAF130*, *SSF2*, *YVH1*), EcoRI/PstI (*FUR1*) or XbaI/HindIII (*MFT1*) and cloned into the plasmid YEplac195 digested with the same set of enzymes [34].

### RNA purification and Northern blot analysis

To prepare control RNA from baker's yeast cells, 0.2 g pieces of compressed yeast blocks were homogenized with 10 ml of ice-cold LETS buffer (200 mM LiCl, 20 mM EDTA, 20 mM Tris-HCl [pH 8.0], 0.4% SDS). Aliquots of 0.5 ml of the cell suspension were transferred to screw-cap microcentrifuge tubes containing 0.5 ml of phenol and 0.5 ml of glass beads (acid-washed, 0.4-mm diameter). Then, the suspension was mixed vigorously 2 times for 45 s each time in a FastPrep device (Bio101). RNA was purified as previously described [11]. Cells from LD were harvested by centrifugation, resuspended in 0.5 ml of LETS buffer and treated as above.

Equal amounts of RNA (30 μg) were separated in 1% (w/v) agarose gels, containing formaldehyde (2.5% v/v), transferred to a Nylon membrane and hybridized with nonradioactive DIG labeled probes containing sequences of *PIS1 *(+41 to +571), *PHO3 *(+1 to +1,381),*OLE1 *(+112 to +1,106), *HSP12 *(+1 to +311) and *HSP26 *(+51 to +508). Oligonucleotides for synthesis of probes are reported in additional file 3. DNA sequences were obtained from the MIPS database (available at http://mips.gsf.de). PCR labeling of DNA probes, membrane pre-hybridizations and hybridizations were performed with the PCR DIG Probe Synthesis Kit and DIG Easy Hyb solution of Roche (Roche Diagnostics GmbH, Mannheim, Germany). After stringency washes, the blots were subjected to immunological detection using anti-digoxigenin antibody conjugated to alkaline phosphatase (Roche), followed by CDP-Star detection (Roche). Images were captured with the Las-1000 Plus imaging system (Fuji, Kyoto, Japan). Spot intensities were quantified with the Image Gauge software version 3.12 (Fuji). Values of spot intensity were evaluated with respect to the rRNA level and represented as the folds of induction/repression of each mRNA.

### Synthesis of target cDNA and DNA filter hybridization

Labeling by random priming using [α-^33^P]dCTP (3,000 Ci/mmol; 10 μCi/μl) was performed as described [35]. The labeled cDNAs were purified by using a MicroSpin S-300 column (Amersham Biosciences). Between 3 × 10^6 ^and 5 × 10^6 ^dpm/ml of labeled cDNA was used for filter hybridization. The purified target cDNA was kept at 4°C until used. Prehybridization, hybridization, and washing were carried out according to published protocols [35]. Replicate samples were prepared from three independent experiments of both high-sucrose and lean LD fermentation.

### Data generation, correction, and normalization

Yeast macroarrays containing the whole genome (6,052 ORFs) of yeast strain FY1679 on 7.5 × 11-cm nylon filters, were provided by the DNA chips laboratory of the University of Valencia (SCSIE, Valencia, Spain, http://scsie.uv.es/chipsdna). The macroarrays have been constructed and the protocols for their use have been evaluated as previously described [35]. A total of 3 different nylon filters were used (one for each experimental replicate). cDNA labeled samples belonging to the same experimental replicate were successively hybridized against the same filter. Therefore, each determination is obtained from the averaged results of three independent filters. Previous studies have shown that the same membrane can be used up to 12 times with satisfactory results [35]. Hybridization signals were quantified using ArrayVision 7.0 software (Imaging Research, Inc., St. Catharines, Ontario, Canada), taking the artifact-removed median density (with the corresponding subtracted background) as signal. Poor or inconsistent signals were not considered for further analysis. The use of the same DNA chip for successive cDNA hybridization improved the comparisons between values for each gene. cDNA hybridizations were normalized within each experiment replicate by the global mean procedure. Reproducibility of the replicates was tested by ArrayStat software (Imaging Research, Inc.), considering the data as independent and allowing the program to take a minimum number of two valid replicates in order to calculate the mean and standard deviation values for every gene (only one of the three replicates was allowed to be a removable outlier). To detect differentially expressed genes, a z-score was obtained for every gene. A *p*-value of 0.05 and a median false discovery rate of 0.5% were used. The change in expression level of a transcript is considered when the log_2 _ratio is equal to ± 2 (fold change of 4).

### Functional category searches

Statistical assessment of overrepresentation of GO biological processes categories [36]http://www.geneontology.org/ among significantly changed sets of transcripts was achieved using the Database for Annotation, Visualization and Integrated Discovery (DAVID) 2006 [37], which is available at http://david.niaid.nih.gov. The *Saccharomyces *Genome Database http://www.yeastgenome.org and the MIPS Comprehensive Yeast Genome Database http://mips.helmholtz-muenchen.de/genre/proj/yeast/ were used to retrieve information about specific gene function and biological process. The list of *S. cerevisiae *ESR genes was obtained from the Web site of Gasch *et al*. [6], at http://www-genome.stanford.edu/yeast_stress. The data of deletions resulting in high-sucrose sensitivity was obtained from Ando *et al*. [13].

## Competing interests

The authors declare that they have no competing interests.

## Authors' contributions

RPT assisted with data analysis and interpretation, statistical analysis and manuscript writing. JP carried transcriptomic experiments. MJHL performed the Northern blot experiments. JAP conceived the study, participated in its design, and contributed to the writing of the manuscript. FRG constructed the strains, performed fermentative capacity experiments, conceived the study, participated in its design, and writing of the manuscript. All authors read and approved the final manuscript.

## Supplementary Material

Additional file 1 **Comparison of Northern blots versus gene filter data**. The fold-change in expression level of five marker genes, *PIS1*, *PHO3*, *OLE1*, *HSP12 *and *HSP26*, as obtained by Northern blot (white bars) or gene filters (black bars) was compared. Total RNA extraction from cells of the L'Hirondelle strain and global gene expression analysis were performed as described in the Materials and Methods section. Cells from compressed yeast blocks (time zero) were used as control. Click here for file

Additional file 2 **List of regulated genes after 60 min of transfer of baker's yeast cells to lean or high-sucrose liquid dough**. Genes specifically up- or down-regulated in lean liquid dough (LLD) and high-sucrose liquid dough (HSLD), and those commonly up- or down-regulated are shown. Expression values showing more than 4-fold induction or repression (log2 ratio of ± 2) are colored in red and green, respectively. Each value indicates the ratio of the level of expression in liquid dough-transferred cells at 60 min to that in control cells from compressed yeast blocks. Click here for file

Additional file 3 **Oligonucleotides used in this study**. Sequences of forward and reverse primers employed to amplify by PCR the mentioned genes. Click here for file
