# Supplementary figures and images for: Global expression studies in baker's yeast reveal target genes for the improvement of industrially-relevant traits: the cases of CAF16 and ORC2
Source: Microb Cell Fact. 2010 Jul 13;9:56. doi: 10.1186/1475-2859-9-56 (PMC2912791; doi:10.1186/1475-2859-9-56)

**A**

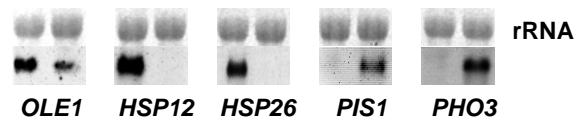

**B**

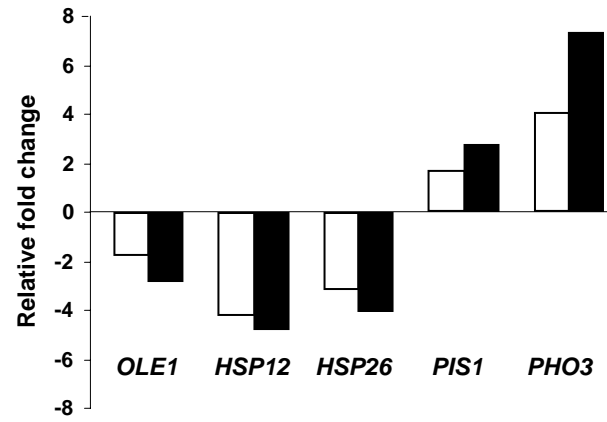

Supplement: Additional file 1 — Comparison of Northern blots versus gene filter data. The fold-change in expression level of five marker genes, PIS1, PHO3, OLE1, HSP12 and HSP26, as obtained by Northern blot (white bars) or gene filters (black bars) was compared. Total RNA extraction from cells of the L'Hirondelle strain and global gene expression analysis were performed as described in the Materials and Methods section. Cells from compressed yeast blocks (time zero) were used as control. [file 1475-2859-9-56-S1.PDF]
