# Supplementary material for: Global expression studies in baker's yeast reveal target genes for the improvement of industrially-relevant traits: the cases of CAF16 and ORC2
Source: Microb Cell Fact. 2010 Jul 13;9:56. doi: 10.1186/1475-2859-9-56 (PMC2912791; doi:10.1186/1475-2859-9-56)
Supplement: Additional file 3 — Oligonucleotides used in this study. Sequences of forward and reverse primers employed to amplify by PCR the mentioned genes. [file 1475-2859-9-56-S3.PDF]

Oligonucleotides used in this study

Primer	Sequence 5´ to 3´	Comments
CAF16-1	GATTTTGACCTGCAGCCATG	Construction of the *CAF16* expression cassette
CAF16-2	GTGCATTTGCATGAACTC	
CAF130-1	CATGTATGTCGACGATCGTG	Construction of the *CAF130* expression cassette
CAF130-2	GATCGAAAAGCTTTTTATAG	
CDC10-1	CTTGTTGTAGCTTTTCTCTC	Construction of the *CDC10* expression cassette
CDC10-2	CAAACTTTTATTCTAGAATTAC	
FUR1-1	AGGGCCAAGAATTCTCGGAA	Construction of the *FUR1* expression cassette
FUR1-2	TATAGCCCCTGCAGAAAGAA	
MFT1-1	CTCAGGGTCAGACACTTCC	Construction of the *MFT1* expression cassette
MFT1-2	GACCAGAGAAGCTTCAAAAC	
NMT1-1	CCTCATTATCTGCAGTTTCC	Construction of the *NMT1* expression cassette
NMT1-2	GTGAGTATCTAGACCAGAAGAG	
ORC2-1	CTCTTTCTGCAGGAACTCTC	Construction of the *ORC2* expression cassette
ORC2-2	TGGAGTGTACCTTACCG	
SEC14-1	AAACACAGCCGGCGTCCATT	Construction of the *SEC14* expression cassette
SEC14-2	AGGTATGGCTGCAGAAAATG	
SSF2-1	CAATCAAGATTAACGCCAAA	Construction of the *SSF2* expression cassette
SSF2-2	GTAAAAGCTTTCTATATATTTG	
YVH1-1	GCTACCAGTCGACAACAGAG	Construction of the *YVH1* expression cassette
YVH1-2	CGACGGAGAAGCTTCAAAGG	
ZUO1-1	CCAATGGCTGCAGGAACTTG	Construction of the *ZUO1* expression cassette
ZUO1-2	GACACCTTTGATCTTTCGTT	
HSP26-1	AGTTGATGCCTTTAACAG	Northern blot. Probe of *HSP26*
HSP26-2	AATGTTGTCTGCATCCAC	
HSP12-1	ATGTCTGACGCAGGTAGAAA	Northern blot. Probe of *HSP12*
HSP12-2	TTACTTCTTGGTTGGGTCTT	
OLE1-1	GCTACTGGTTTGAATAAG	Northern blot. Probe of *OLE1*
OLE1-2	TTCAAGTCGTATGCTAGA	
PIS1-1	ACGTTCTGTGGTATATTC	Northern blot. Probe of *PIS1*
PIS1-2	TGGACAACGTTTGCGGTCTG	
PHO3-1	ATGTTTAAGTCTGTTGTTTAT	Northern blot. Probe of *PHO3*
PHO3-2	TGTTTTAATAGGGTATCGTT	
